# Polyphasic Approach and Potential Cyanotoxin Production by *Planktothrix* from the Río Grande de Comitán and Montebello Lakes National Park, Southern Mexico

**DOI:** 10.1155/2024/9993635

**Published:** 2024-05-11

**Authors:** Javier Carmona Jiménez, Angela Caro Borrero, Aída Isabel Sánchez-Salas, Itzel Becerra-Absalón, Samuel Cirés Gómez, Antonio Quesada del Corral, Elvira Perona Urizar, David Ortíz Suárez, Marisa Mazari-Hiriart

**Affiliations:** ^1^River Ecosystem Laboratory, Department of Ecology and Natural Resources, Faculty of Science, National Autonomous University of Mexico (UNAM), Exterior Circuit, University City, Coyoacan, Mexico City 04510, Mexico; ^2^Postgraduate Program in Marine Science and Limnology, UNAM, Exterior Circuit, University City, Coyoacan, Mexico City 04510, Mexico; ^3^Laboratory of Ficology, Department of Comparative Biology, Faculty of Sciences, UNAM, Exterior Circuit, University City, Coyoacan, Mexico City 04510, Mexico; ^4^Department of Biology, Faculty of Sciences, Autonomous University of Madrid (UAM), C/Darwin 2, Cantoblanco, Madrid 28049, Spain; ^5^Department of Chemical Engineering, Faculty of Sciences, UAM, C/Francisco Tomás y Valiente 7, Cantoblanco, Madrid 28049, Spain; ^6^National Laboratory of Sustainability Sciences, Institute of Ecology, UNAM. Exterior Circuit, University City, Coyoacan, Mexico City 04510, Mexico

## Abstract

The development of anthropic activities during recent years has led to an increase in nutrient fluxes in the Río Grande de Comitán and Montebello Lakes National Park, Mexico. In turn, this has modified the dynamics of the biotic community, specifically favoring the presence of cyanobacteria tolerant to contamination. The continual and massive presence of *Planktothrix* species (spp.) in the system suggests a potential detrimental impact for economic issues and human health. In this study, we identify the morphological and molecular characteristics of *Planktothrix* populations from seven tropical (1,380–1,740 masl, 23.0–25.5°C) and calcareous lakes and two ponds from a water treatment plant. We also assess the ecological drivers that could be related to the presence of cyanotoxins in the system. The ecological preferences, morphology, 16S rRNA structure, and 16S-23S rRNA internal transcribed spacer found evidence for three species: *P. agardhii* distributed in neutral to slightly basic water (pH = 7.7–8.7), and *P. spiroides* and *Planktothrix* sp. in alkaline waters (pH = 9.1). The presence of the *mcyE* gene and its validation by liquid chromatography confirmed the presence of two microcystin variants (MC-RR and MC-LR) in at least three populations of *P. agardhii*. These microcystins put the health of the ecosystem and its inhabitants at risk, a condition that should be addressed and resolved with a water management and detoxification strategy in the basin.

## 1. Introduction

Cyanobacteria of the genus *Planktothrix* Anagnostidis et Komárek have been cited as abundant and frequent components in freshwater lake communities, known to form cyanobacterial blooms in temperate, tropical, and boreal climates throughout the world [1, 2]. The genus is characterized by trichomes that are solitary, rarely in small, irregular, and easily disintegrating fascicles (groups) (mainly in blooms), straight and isopolar, with aerotopes through the whole cell volume, and the end cells widely rounded or slightly narrowed. The systematic position of *Planktothix* species has been controversial due to morphological similarities and overlapping morphometric variation given the morphological plasticity within the genus [1, 3]. Molecular evidence using 16S rRNA sequences was a major contribution to delimiting and separating the *Planktothix* genus from *Oscillatoria* [4]. From morphological and molecular evidence, 21 species are currently recognized in the genus worldwide, but controversy still surrounds five species [5]. Suda et al. [3] established a new genus *Planktothricoides*, composed of the type species *Planktothricoides raciborskii* (originally *Planktothrix raciborskii*), as well as a new species *Planktothrix pseudagardhii*, two genera that are clearly delimited in genetic terms.

*Planktothrix* populations have the ability to produce secondary metabolites, including cyanotoxins, microcystins (MCs), a group of hepatotoxins whose ecological role is not well understood [6]. According to the World Health Organization (WHO) [7], prolonged exposure to MCs produces phosphatase inhibition, which, in humans, induces liver hypertrophy and tumor-promoting activity. For this reason, international regulations established that the permissible limit for MCs (estimated based on the most common variant) in drinking water must be less than 1 *μ*gL^−1^ [8]. Nutrient concentrations, as well as the parameters that promote the synthesis of MCs, are essential for assessing the potential presence of cyanobacterial blooms, particularly in *Planktothrix* populations [9–11]. For example, Gagała et al. [12] reported blooms of *P. agardhii* (Gomont) Anagnostidis et Komárek having concentrations of total nitrogen (TN) above 1.5 mg/dm^3^, total phosphorus (TP) greater than 0.1 mg/dm^3^, and a TN : TP ratio of 29 : 1, in temperatures 18–30°C and in a pH of 6–9. Catherine et al. [13] and Teubner et al. [14] argued that massive growth is promoted at ratios of 16 TN : 1 TP. Other studies have revealed significant growths at low TN concentrations (0.62–0.74 mg/L) and soluble reactive phosphorus (SRP) levels between 1 and 20 *µ*g/L [9]. However, some studies in lakes have shown that even when waters have recovered to mesotrophic or oligotrophic levels, blooms persist, suggesting that other ecological factors also play an important role [15]. With respect to factors affecting toxin production, Van de Waal et al. [16] found that an increase in TN is related to MC-RR synthesis.

In Mexico, *P. agardhii* has been previously reported in hypereutrophic lakes. Komárek and Komárková-Legnerová [17] and Pineda-Mendoza et al. [18] analyzed the taxonomic and molecular characteristics of *P. agardhii* in Xochimilco and Chapultepec urban lakes in Mexico City. Likewise, Vasconcelos et al. [19] reported this species in the Valle de Bravo dam and detected MCs using ELISA. According to Vasconcelos et al. [19], visible blooms of *P. agardhii* were present in Cuemanco, Mexico City, an artificial water channel used for sports and recreational activities. Molecular and chemical analyses found MCs in Cuemanco to be 4.9 *µ*g MC-LR eq/L. This concentration is comparable to those found in many countries where a potential risk to human health has been confirmed [20–24]. In tropical areas of the Mexican southeast, the presence of cyanobacteria has been frequently reported in the Montebello Lakes National Park, where blue-green waters with different color intensities are commonly observed [25]. The detection of MC-LR in three lakes by ELISA immunoassays was found to be related to the presence of *Limnothrix*, *Planktothrix*, and *Raphidiopsis* spp. as potential producers of the toxin [26]. The lake area of the national park is subject to anthropogenic pressures such as tomato cultivation, which increases the supply of nutrients to the lakes. In addition, the area supplies water to adjacent towns and bears the impact of a growing tourism industry. While these studies represent important contributions to our understanding of cyanobacterial blooms, very few studies in Mexico have characterized the structures of these blooms at a biological and ecological level to determine the causes of their proliferation and their potential effects on the ecosystem. In this sense, the aim of this study is to identify the morphological and molecular characteristics of recurrent populations of *Planktothrix* and the ecological disturbances that could be related to the presence of cyanotoxins in the Río Grande de Comitán sub-basin and Montebello Lakes National Park, Mexico.

## 2. Material and Methods

**Figure 1:**
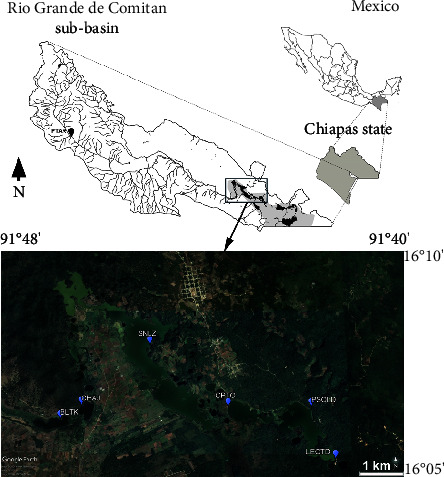
Sampling sites of *Planktothrix* populations in six natural lakes and one sedimentation pond in a water treatment plant (PTAR) within the Río Grande de Comitán sub-basin and Montebello Lakes National Park, Mexico. Water treatment plant (PTAR), Balamtetik (BLTK), Chaj Chaj (CHAJ), San Lorenzo (SNLZ), Chilpotrero (CPTO), Paso del Soldado (PSOLD), Bosque Azul (BAZUL), and La Encantada (LECTD).

The Río Grande de Comitán sub-basin is located in the National Hydrological Region No. 30 Grijalva-Usumacinta, State of Chiapas [27]. It lies between 16°04′40″–16°10′20″ LN and 91°37′40″–91°47′40″ LW and spans an elevation of 1,380−1,740 masl. The climate is characterized as temperate humid and warm humid, with abundant rains in the summer [28]. The main tributary of the sub-basin is the Río Grande, which runs through urban and agricultural areas until it flows into the Montebello Lakes National Park (Figure 1). The karst origin of the region promotes the circulation of underground water that communicates with a complex system of 59 lagoons [29]. This area is of great ecological, cultural, and economic importance for the region, leading to it becoming established as a protected natural area by presidential decree published in the Diario Oficial de la Federación on December 16, 1959, and later designated as a RAMSAR site in 2003. Since 2003, however, color changes have been observed in the lakes, reportedly related to the increase in phytoplankton biomass as a consequence of the increasing eutrophication of the lakes [26, 30, 31]. The human communities surrounding these lakes utilize the water for both direct use and consumption, as well as for agricultural and silvopastoral activities [28, 32].

### 2.1. Ecological Characterization

Fieldwork was performed during the rainy season (August 2019), corresponding to the most favorable growth period for cyanobacterial populations in the region [25], in six natural lakes and one sedimentation pond in a water treatment plant from the Río Grande de Comitán sub-basin and Montebello Lakes National Park (Table 1). The sampled lakes were selected based on the presence of blue-green coloration as a possible indicator of cyanobacterial blooms. Temperature, pH, specific conductance, dissolved oxygen, orthophosphate, ammonium, and nitrates were measured for each reservoir with YSI electrodes (Ohio, USA). We sampled one liter per site to monitor water quality and health hazards of recreational waters and the accumulation of algal matter at the downwind end of the lake or shore. The sampled water was kept cold (4°C) for algal isolation and subsequent cultivation in the laboratory. Later, algal samples were preserved in 4% formalin to preserve the useful cytological structures for taxonomic identification. Abundances of each morphospecies were estimated in quadruplicate subsamples in a Neubauer chamber (depth of 0.1 mm and 0.0025 mm^2^ in area) and calculated using the following equation: Abundance (cel/ml) = amount of cells/number of quadrants × 10,000.

To determine water quality in the lakes, one-liter samples of water were collected in sterile polypropylene flasks for bacteriological analyses, stored at 4°C, and processed within 24 h of collection using the membrane filtration technique [33]. Membrane filters (cellulose acetate, 0.45 *μ*m, Millipore MF type HA) were placed in Petri dishes with 2.5 mL of membrane fecal coliform agar medium and incubated at 35°C for 24 h and with Kenner fecal Streptococcus agar for fecal Enterococci and incubated at 44.5°C for 48 h [33].

### 2.2. Morphological Characterization

For each site sampled, three laminas were observed and their abundance quantified with a Neubauer chamber according to Hötzel and Croome [34]. Specimens collected within sites were measured to determine the morphometric characteristics determined previously to be of taxonomic importance: length and thickness of trichomes, and width and length of vegetative cells [1, 2]. Measurements of morphometric characteristics were made in the most abundant populations collected (Balamtetik, Chaj Chaj, and water treatment plant) in replicates for 60 individuals. Differences in morphometric characters between populations were evaluated by one-way analysis of variance (ANOVA, *p* ≤ 0.05) followed by Tukey's tests to know which means are statistically different from each other. Tests were performed with the STATISTICA v. 3.0 statistical package.

### 2.3. Molecular Characterization (16S rRNA Gene)

Molecular analyses were carried out on the five samples with the highest cell concentrations (Table 1). DNA extractions were performed using the QIAGEN DNeasy UltraClean Microbial Extraction Kit following the manufacturer's protocol. The field samples were concentrated with GF/F filters and pretreated to facilitate cell rupture, which consisted of three freeze/thaw cycles with liquid nitrogen and heating on an AccuBlock (Labnet International Inc.) at 65°C. Between each cycle, a drill and plastic pistil were used. The presence of DNA was confirmed by electrophoresis (0.8% agarose gel), and DNA concentration was measured using a microplate spectrophotometer (Epoch: BioTek Instruments Inc., USA).

Amplification of the 16S rRNA gene was performed by PCR using Biometra Tone Thermal Cyclers (Analytik Jena, Göttingen, Germany). The following reaction master mix was used: milli-Q water, 10x PCR buffer, Cl_2_Mg (50 mM), deoxyribonucleotide triphosphate (50 *μ*M dNTP), bovine serum albumin (BSA, 0.1%), DNA polymerase (Ultratools DNA Polymerase: 1 unit/*μ*L, and Thermo Scientific DreamTaq DNA Polymerase: 20 and 500 units/*μ*l). The primers (10 pM) used include the oligonucleotides 27F (5′-AGAGTTTGATCCTGGCTCAG-3′) [35] and 23Sr (5′-CTTCGCCTCTGTGTGCCTAGGT-3′) [36]. Subsequently, an agarose gel electrophoresis (1.5%) was performed to reveal the PCR product. Once the DNA bands were obtained, the PCR product was purified using the Wizard SV Gel and PCR Clean-up System Kit (Promega). Subsequently, the cloning procedure using the pGEM-T Easy Vector System Ligation Kit (Promega) was carried out to ensure the greatest obtention of copies of the genetic material and the greatest representativeness of the algal community. The transformation process was carried out using 100 *µ*L of the competent bacteria strain *Escherichia coli* (DH5a) (Promega). The transformed bacteria were inoculated (250 *μ*L) in Petri dishes with solid LB medium [37], ampicillin (0.1 mg/mL), X-Gal: 5-bromo-4-chloro-3-indolyl-*β*-D-galactopyranoside (0.04 mg/mL), and IPTG: isopropyl-*β*-D-1-thiogalactopyranoside (0.5 mM) [38]. The cultures were incubated at 37°C for 24 hours. Subsequently, the positive clones (presence of the insert) were recognized by their white coloration and reseeded in LB medium. Negative clones (blue colonies without insert) were discarded. The presence of the insert was confirmed by PCR and electrophoresis [38]. The reaction master mix used was the following: milli-Q water, 10x PCR buffer, Cl_2_Mg (50 mM), deoxyribonucleotide triphosphate (50 *μ*M dNTP), and DNA polymerase (Ultratools DNA Polymerase: 1 unit/*μ*L and Thermo Scientific DreamTaq DNA Polymerase: 20 and 500 units/*μ*l). As primers (10 pM), the oligonucleotides T7 (5′-TAATACGACTCACTATAGGG-3′) and SP6 (5′-ATTTAGGTGACACTATAG-3′) [39] were used.

Once the presence of the insert was confirmed, the transformed bacteria were cultured in liquid LB medium with ampicillin at 37°C and horizontal movement of 250 rpm for 24 hours. Afterward, the extraction and purification of the plasmids was carried out using the Wizard Plus SV Minipreps DNA Purification System Kit (Promega). Once the plasmid DNA was obtained, its concentration was measured (Epoch: BioTek Instruments Inc., USA), before finally be sent for sequencing [38]. The sequencing process was carried out at the DNA Synthesis and Sequencing Unit (USSDNA) of the Institute of Biotechnology-UNAM (National Autonomous University of Mexico) and the Complutense University of Madrid (Genomic Unit-CAI). The primers used for sequencing were T7 (5′-TAATACGACTCACTATAGGG-3′), SP6 (5′-ATTTAGGTGACACTATAG-3′) [39], and 684F (5′-GTGTAGCGGTGAAATGCGTAGA-3′) [40].

#### 2.3.1. Taxonomic Identification from the Sequences Obtained

The sequences obtained were assembled using the BioEdit 7.2 program [41] to obtain consensus sequences, before a subsequent BLAST analysis (Basic Local Alignment Search Tool: https://blast.ncbi.nlm.nih.gov/Blast.cgi). Sequences with identity percentages above 98% similarity were considered. Together with the results of the morphological identification, a taxonomic identity was obtained for the populations of cyanobacteria analyzed. A phylogenetic tree was built with the sequences obtained for each of the study populations, incorporating similar sequences identified by the BLAST analysis and additional sequences of some genera pertaining to closely related taxonomic groups (https://www.cyanodb.cz/ and https://www.ncbi.nlm.nih.gov/). Once the sequence matrix had been constructed, a multiple alignment was performed using the BioEdit program, followed by a manual revision in the PhyDE-1 V 0.9971 program [42]. Manual alignment was performed under the criterion of maximum parsimony. Next, phylogenies were constructed in the MEGA V.7.0.26 program [43], using the following algorithms: (1) Neighbor Joining, (2) Maximum Parsimony, and (3) Maximum Likelihood. To confirm the genetic variation between species, the RNA-ITS region was analyzed. Sequences from the rRNA-ITS region of *P. rubescens* (De Candolle ex Gomont) Anagnostidis et Komárek, *P. paucivesiculata* Gaget, Welker, Rippka et de Marsac, *P. agardhii*, and *P*. *pseudagardhii* Suda et Watanabe were obtained from GenBank and aligned using the ClustalW multiple alignment program in BioEdit. The analysis was performed in the online program CIPRES (phylo.org) using the PAUP tool on XSEDE, under default parameters.

### 2.4. Isolation and Strains

To confirm the genetic identity and toxicity of *Planktothrix* populations, samples were isolated from three sites within the study area: Bosque Azul, San Lorenzo, and Paso del Soldado, on July 10, 2023 (Table 1). Unialgal trichomes from the cyanobacterial samples were isolated using a Pasteur pipette under the Olympus SC31 microscope (Japan) and cultured in Petri dishes containing BG11-agar medium. All isolates were subsequently cultivated at 20°C under a 12 : 12 h (light : dark) cycle with a photon flux density of 40–45 *μ*mol photons m^2^ s^−1^ from white fluorescent lamps. Living cultures were maintained in the laboratory culture collection at the Universidad Autónoma de Madrid, Spain.

#### 2.4.1. Cyanotoxin Gene Detection and Chemical Characterization

To screen for the presence of MC biosynthesis gene clusters, the peptide synthetase-encoding gene *mcyE* was selected as a target. The following reaction master mix was used: milli-Q water, 10x PCR buffer, Cl_2_Mg (50 mM), deoxyribonucleotide triphosphate (50 *μ*M dNTP), and DNA polymerase (QIAGEN DNA Polymerase: 1 unit/*μ*L and Thermo Scientific DreamTaq DNA Polymerase: 20 and 500 units/*μ*l). As primers (10 pM), the oligonucleotides HEPF (5′-TTTGGGGTTAACTTTTTTGGGCATAGTC-3′) and HEPR (5′-AATTCTTGAGGCTGTAAATCGGGTTT-3′) [44] were used.

The PCR products were checked by agarose gel electrophoresis (1.5%), and the purification and cloning of amplified DNA fragments followed previously described procedures. To determine the DNA concentration of the samples, plasmid DNA obtained from the cloning was measured using a NanoDrop UV spectrophotometer. The genes were sequenced on a Sanger 3730xl Sequencer (Thermo Fisher Scientific, MA, USA). Partial sequences were compared to those available in the NCBI database using BLASTn, while the BLAST X tool (blast.ncbi.nlm.nih.gov/Blast.cgi) was used for *mcyE* gene.

Toxins were extracted from 50 mL culture samples and filtered through Whatman GF/F filters (Whatman International Ltd., Brentford, UK) until saturation and stored at −20°C until extraction. Microcystins were extracted twice with 90% methanol and concentrated by evaporation at 50°C under vacuum.

High-performance liquid chromatography (HPLC) detection of MC-LR and MC-RR microcystin variants was analyzed using HPLC-UV (Shimadzu, mod. Prominence-i, LC-2030C LT) and a diode array detector (SPD-M30A). An Eclipse Plus C18 column (150 × 46 mm, 5 *µ*m) (Agilent) was used as the stationary phase, while mobile phases were characterized by a mixture of 37/63, 30/70% (v/v) of acetonitrile and acetic acid aqueous solution (75 mM) for the MC-LR and MC-RR analyses, respectively. The temperature of the column was fixed at 35°C, and the mobile phase flow rate was fixed at 0.8 mL/min. Both analyses were carried out at 239 nm for both MC-LR and MC-RR. Microcystin concentrations (ppb or ng/ml) were calculated from the area under the curve that was observed at retention times coinciding with the standard substance. This concentration reflects the intracellular amount of MCs in the cyanobacterial cultures analyzed.

## 3. Results

### 3.1. Ecological Characteristics

According to physical and chemical measures, the study sites showed the following environmental characteristics: temperate waters (23–25.5°C), mostly neutral to alkaline pH (7.9–9.1), and variable oxygen concentration (3.4–10.6 mg/L). In general, orthophosphate (0.1–38.3 mg/L), ammonium (0.07–6.71 mg/L), and nitrate (0.81–25 mg/L) concentrations were high, and the presence of fecal *Enterococci* was recurrent in all water bodies, probably related to the urban and agricultural activities around the sampled areas (Table 2).

**Figure 2:**
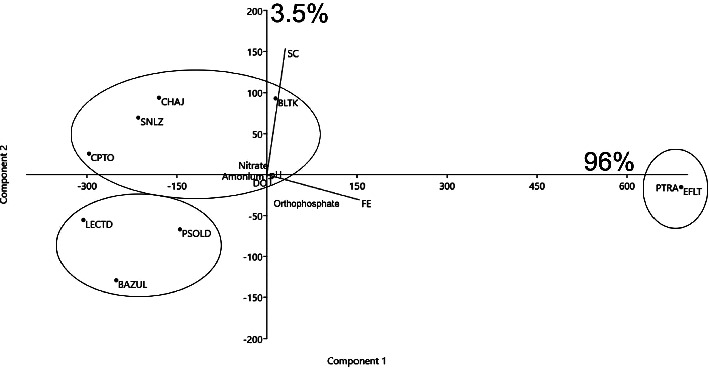
Site groups based on the PCA of physical, chemical, and bacteriological parameters in the water treatment plant and lakes of the Río Grande de Comitán sub-basin and Montebello Lakes National Park.

Based on the PCA (Figure 2), the sites can be classified into three main groups: (1) the first group (the water treatment plant and Efluente) is classified as polluted sites with high fecal Enterococci (0.98), associated with the first component explaining 96.4% of the total variance; (2) the second group is composed of four sites (Balamtetik, Chaj Chaj, San Lorenzo, and Chilpotrero) characterized by high specific conductance (0.97) and associated with the second component explaining 3.5% of the total variance; these sites could represent transitional conditions, as they do not show a positive correlation with nutrient increase; and (3) the third group is represented by three sites (Paso del Soldado, Bosque Azul, and La Encantada) that are characterized by decreased levels of orthophosphate, ammonium, nitrate, and fecal Enterococci.

The abundance of *Planktothrix* populations showed a similar relationship to the three groups of water bodies recognized by the ecological PCA. The greatest abundance of *Planktothrix* was present in the treatment plant, followed by Balamtetik, Chaj Chaj, and San Lorenzo, while the lowest abundance was observed in Chilpotrero, Paso del Soldado, and La Encantada (Table 2).

### 3.2. Morphological Analysis

**Figure 3:**
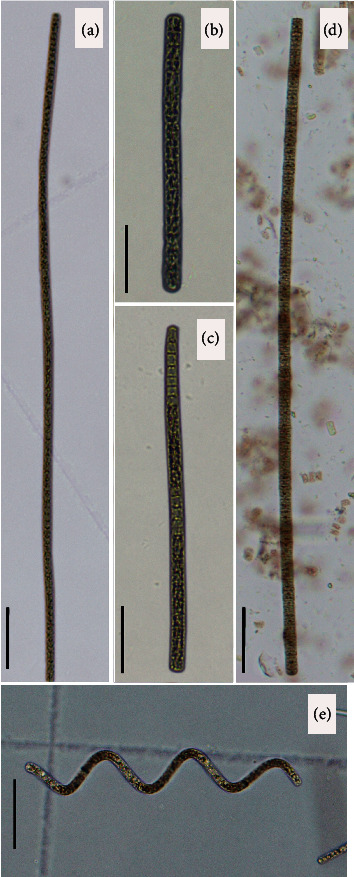
Morphological characteristics of *Planktothrix* populations in the Río Grande de Comitán sub-basin and Montebello Lakes National Park. *Planktothrix agardhii*: (a) Chilpotrero, (b) San Lorenzo, and (c) Chaj Chaj. Planktothrix sp: (d) Efluente. *P. spiroides*: (e) Water treatment plant. Scale bar = 10 *µ*m.

In the eight bodies of water collected for the present study, mostly solitary trichomes, or formed groups of trichomes, were recorded as free-floating in eutrophic conditions. The trichomes were long (up to 420 *µ*m), straight, without sheaths, immotile, not very constricted at the granulated cross-walls, and 2.69–5.57 *µ*m wide. Cell contents were blue-green colored, with a few big aerotopes. Apical cells were convex with calyptra. These morphological characteristics identify the species as *Planktothrix agardhii* (Figures 3(a)–3(c)).

The Tukey test revealed significant differences for relative trichome length, cell length, and cell diameter (*p*=0.001–0.008) between populations (Table 3).

The ANOVA test found three groups of populations recognized based on cell dimensions (*F* = 24.92-27.23, *p* < 0.05). The first group, from the treatment plant, has cells characterized by having greater cell width (mean = 5.2 *µ*m) but shorter in length (mean = 2.0 *µ*m). The second group, from La Encantada, is characterized by a shorter cellular length (mean = 3.6 *µ*m) but greater cell width (mean = 3.8 *µ*m). The third group, from Efluente, Chaj Chaj, Balamtetik, San Lorenzo, Chilpotrero, and Paso del Soldado, exhibits cellular dimensions that are intermediate between those recorded in the first two groups. There were important overlaps in the trichome lengths between the populations, but the longest trichomes were recorded in San Lorenzo and Chilpotrero (mean = 231.7–245.4 *µ*m), while smaller trichomes were observed in the other populations (mean = 109.13–189.39 *µ*m).

In the water treatment plant, spiral trichomes were recorded in a free-floating bloom (Figure 3(e)). Trichomes were short (<89.8 *µ*m), lacking sheaths, not constricted across walls, rarely motile, with cylindrical cells 3.0–6.0 *µ*m wide, usually shorter than wide, and with mucilaginous sheaths rare. Cell contents were olive-green in color, with several small gas vesicles distributed throughout the cells. The apical cells were rounded to be slightly attenuated, without calyptra. These morphological characteristics key to *P*. *spiroides*.

A different morphotype was observed in the Efluente site. Here, the trichomes were short (<83.15 *µ*m), straight, lacking sheaths, slightly constricted across walls, and 3.3–6.4 *µ*m wide. Their cell contents were olive-green in color, with several small aerotopes. Apical cells were convex, without calyptra (Figure 3(d)). These morphological characteristics fail to key to any described species, suggesting that this population may represent a new species, but more taxonomic studies are necessary to confirm this.

### 3.3. Molecular Analyses

**Figure 4:**
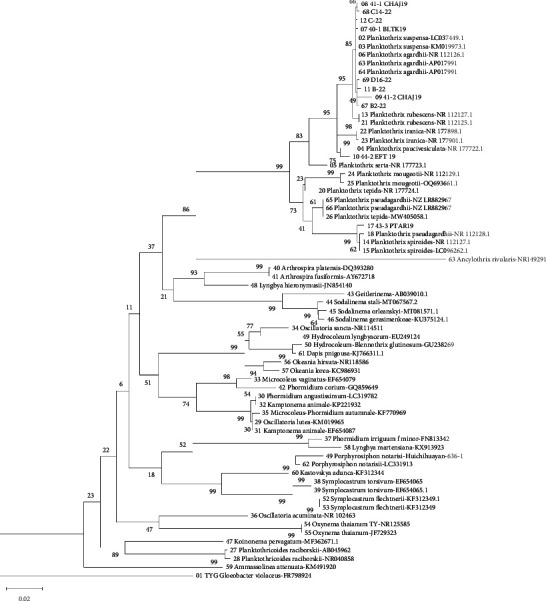
Bayesian inference analysis of 16S rRNA sequences of samples from this study combined with other samples of the family Microcoleaceae obtained from NCBI. Bootstrap values are shown at the nodes and based on 1000 resamplings. The light gray square represents *Planktothrix agardhii*, the dark gray square *P. spiroides*, and the white square *Planktothrix* sp.

The DNA sequencing of the clones obtained (Figure 4) corroborated the presence of *P. agardhii* and *P. suspensa* in Balamtetik (40-1BLTK19), Chaj Chaj (41-1 CHAJ19 and 41-2 CHAJ19), Bosque Azul (B-22 and B2-22), and San Lorenzo (C14–22 and C-22), which all form a clade with a bootstrap support value of 85%. Based on the ITS analysis, these samples are identified as *P. agardhii*, with values ≤3%. This result is also corroborated by the morphological description (Table 4). The sample from Efluente (44-2 EFT19) was found to be closely affiliated with *P. paucivesiculata* (BS = 99%) based on the ML tree. However, the ITS analysis shows a clear difference between these two samples, with a value of 6.7%. Based on these results, this population could represent a new species, and further analyses are necessary (e.g., ultrastructural and genetic confirmation in field and strain samples).

### 3.4. Toxin Results

**Figure 5:**
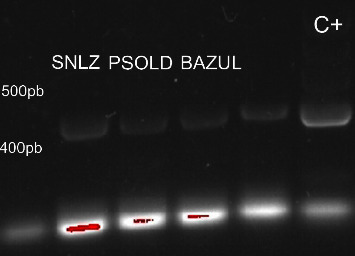
Electrophoresis gel with PCR products for the identification of the *mcyE* gene that is involved in microcystin biosynthesis in *Planktothrix agardhii* strains. The samples pertain to three lakes in the Río Grande de Comitán sub-basin and Montebello Lakes National Park. BAZUL = Bosque Azul, SNLZ = San Lorenzo, PSOLD = Paso del Soldado. C+ = Positive control.

The PCR analysis revealed the presence of *mcyE* gene clusters in the strains of *P. agardhii* from Chaj Chaj (Figure 5). HPLC-UV results reveal the presence of MC-RR and MC-LR in all cultures. The prominent variant in the sample was MC-LR, with 2.0–3.169 *μ*g/L. The MC-RR variant registered values at half to one-third the concentration of MC-LR, with a ratio of 1.96 : 3.02 (Table 5).

## 4. Discussion

Three clearly distinct morphotypes were observed in the sampled populations. The first, and more abundant, morphotype is characterized by long, straight trichomes, blue-green cell contents containing a few big aerotopes, and convex apical cells with calyptra. According to Komárek and Anagnostidis [2], these characteristics belong to *Planktothrix agardhii*. The second morphotype is characterized by short, spiral trichomes, cross-walls not constricted, olive-green cell contents with several small aerotopes, and rounded to be slightly attenuated apical cells without calyptra. The form of the trichomes, cross-wall type, and aerotopes are distinctly different from the populations identified as *P*. *agardhii*, and according to Liu and collaborators [45], this second morphotype pertains to *P*. *spiroides*. The last morphotype is characterized by short, straight trichomes, cross-walls slightly constricted, olive-green cell contents with several small aerotopes, and convex apical cells without calyptra. The short trichomes, aerotope type, and the absence of calyptra differentiate this population from *P*. *agardhii*, while the straight trichomes, cross-walls type, and convex apical cells differentiate this population from *P*. *spiroides*. This morphotype was not able to be identified from the described species [2, 45–47], suggesting that it may represent a new species.

Morphological variation has been reported as a response to environmental heterogeneity in planktic cyanobacterial populations [3, 48]. While our data showing cellular variation in *Planktothrix agardhii* populations could reflect morphological responses to the conditions they experience, this variation does not exceed 6 *µ*m in cell width, which is reported as a relevant characteristic by Komárek and Anagnostidis [2] for the species.

The spiral shape of the trichomes recorded in the water treatment plant typically corresponds to descriptions of the *Arthrospira*–*Spirulina* group [1], but the degree of coiling is known to vary across environmental parameters or change after extended periods of cultivation [49].

The molecular analyses confirmed the morphological identification of the *P*. *agardhii*-like populations, as well as the *P*. *spiroides*-like population, as these populations formed monophyletic clades with NCBI sequences of *P*. *agardhii* and *P*. *spiroides*, respectively, both with high bootstrap values.

While *Planktothrix* sp. formed a clade with *P*. *paucivesiculata*, the morphology was quite different. According to the description of Gaget and collaborators [46], the trichomes in *P*. *paucivesiculata* are thinner, slightly curved, and may present sheaths, as well as the apical cells being rounded.

The frequent presence and abundance of *P. agardhii* throughout the Río Grande de Comitán sub-basin and Montebello Lakes National Park reflects a species with different ecotypes capable of occupying different niches. This was similarly reported in a study on 11 *Planktothrix* genomes from temperate regions of Europe, which related adaptability to buoyancy capacity, ability to fix atmospheric nitrogen, and unique characteristics of natural products [50].

In recent years, Montebello Lakes National Park has registered an increase in phytoplankton biomass, reflected by evident changes in water color [30, 31, 51]. Likewise, high concentrations of carbon, nitrogen, sulfur, organic matter, and chlorophyll a have also been found in the sediment and seston [52]. The contribution of urban wastewater, the intense agricultural activity, and the use of touristic boats may be related to the water pollution and the dispersal of cyanobacteria in the lake system. The high abundance and broad distribution of *Planktothrix* populations in the lake system may be present throughout different seasons of the year ([25], this study), indicating a relatively resilient component of the phytoplankton community.

The basin presents a natural connection along the lagoon system, with some bodies of water apparently isolated on the surface but connected underground [53]. The sites sampled in the present study registered the presence of *Planktothrix* with different abundances, suggesting a natural dispersal by surface transport, by air carriers such as birds, or via various anthropogenic activities such as tourism walks and/or fishing from boats that are shared between the different lakes and not previously sanitized. Consequently, these activities could be favoring the dispersal of species that would not naturally grow in some of the studied lakes.

The highest representation (biomass) of *P. agardhii* is reportedly associated with autumn, when it tolerates low light intensity and lower temperatures, even in tropical latitude lakes [54–58]. However, this species can prevail in temperate climates or even flourish throughout the year in eutrophic systems [6].

This replacement and/or resilient species characteristic could represent an ecological imbalance, as well as a risk to human health since most of the studied sites are used either for drinking water production or agriculture and recreation purposes. *Planktothrix agardhii* is known for its ability to synthesize toxins under bloom conditions, and in competitive situations, it can also produce allelopathic compounds to partially offset the costs associated with MC production [59]. This has been observed in both temperate and tropical water bodies, as well as in shallow lakes dominated by *P. agardhii* and deep stratified lakes harboring *P. rubescens* [6, 50].

The presence of an MC biosynthetic *mcyE* gene cluster was confirmed in *P. agardhii* via sequencing of the *mcyE* gene and by HPLC-UV analyses. Potentially toxic cyanobacteria possess the MC synthetase *mcyE* gene cluster that is inactive or absent in nontoxic strains. Currently, more than 200 structural variants of MCs have been isolated and characterized [60], with MC-LR being among the most toxic and widespread worldwide. Effects in humans can even cause death. As such, by 2005, six countries (Brazil, the Czech Republic, Poland, Canada, France, and Spain) had adopted some regulatory measures for MCs in terms of drinking water quality, which are based on the World Health Organization's provisional TDI (Tolerable Daily Intake) value for MC-LR in drinking water. Three countries (Germany, Finland, and Italy) did not enact their own regulation, but they stated that in cases of potential risk, they would accept the limits proposed by the WHO [7]. In North America, the Environmental Protection Agency (EPA) recommends Health Advisory (HA) levels at or below 0.3 *μ*g/L in drinking water for children preschool age and younger (i.e., less than 6 years old) [61, 62]. For school-age children through adults, the recommended HA levels for drinking water are at or below 1.6 *μ*g/L for MCs.

Confirmation of *Planktothrix* populations in Mexico via phenetic and genetic analyses has become frequent, showing that these cyanobacteria are becoming abundant (Table 6). The only species recognized for Mexico is *P. agardhii*, which is currently reported in bodies of water from the center of the country that, due to their high altitude and water temperature less than 20°C, present conditions similar to temperate regions [50]. Molecular methods and enzyme-linked immunosorbent assays (ELISAs) for detecting cyanotoxins show the presence of MCs in sites where *Planktothrix* populations exist (Table 6). However, those findings are inconclusive since most of the cited analyses were performed on field samples that may contain other potentially toxic cyanobacteria found in the Río Grande de Comitán sub-basin and Montebello Lakes National Park, such as *Microcystis* spp. [19, 68], *Anabaena* spp. [70], or *Raphidiopsis* spp. and *Limnothrix* spp. ([26], Table 6).

The presence of MCs found in strains collected in this study reveals a risk to human health if the water is to be used for drinking without efficient treatment to remove MCs. Therefore, periodic monitoring in the field is recommended, both in the lake water and in the effluent from the water treatment plant. Another indicator of this risk to human and ecosystem health is the large abundance of these cyanobacteria recorded in the field. Specifically, populations exceeding 25,000 cells/ml were observed in lakes. Values less than 20,000 cells/ml are classified by the WHO [6] as a criterion for notifying the health authorities to start vigilance levels in the water body. Ingestion of MCs may occur through accidental uptake during recreational or occupational activities. In some settings, contaminated food can be a possibly significant source of dietary exposure, including fish and crustaceans collected from lakes, as well as leafy vegetable crops spray-irrigated with water containing cyanobacteria.

The characterization of the morphological and genetic variation, as well as the ecological preferences, of two species of *Planktothrix* in the sub-basin of the Río Grande de Comitán and Montebello Lakes National Park was conclusive in identifying the species and establishing a baseline assessment of the structure and functioning in the phytoplankton community. The presence and abundance of *P. agardhii* is notorious throughout the hydrological system, which is disturbed by intense urban, agricultural, and tourist activity. Two variants of MCs in the lakes that directly receive water from the main channel may be related to an increase in runoff nutrients and organic matter that accumulates from the surrounding landscapes. The presence of MCs puts the health of the ecosystem and its inhabitants at risk and represents a situation that should be addressed and resolved with a water management and detoxification strategy specific to the basin. Identifying the biology and health risks of other potentially toxic cyanobacteria reported in the Montebello Lakes National Park (e.g., *Anabaenopsis* sp., *Microcystis* sp., *Limnothrix* sp., and *Raphidiopsis* sp.) ([25, 26], this study) remains necessary to integrate the cyanobacterial risks into water management strategies for this protected area.

## Figures and Tables

**Table 1:** Type of sample used for molecular analysis and positive toxin extracts of *Planktothrix* populations in the Río Grande de Comitán sub-basin and Montebello Lakes National Park.

Site	Code (clone)	Sample type
*Molecular analysis*		
Balamtetik	40-1 BLTK19	Field
Chaj Chaj	41-1 CHAJ19	Field
	41-2 CHAJ19	
	C22	Strain
Bosque Azul	B22	Strain
	2B22	Strain
Paso del Soldado	D16	Strain
Water treatment plant	44-2 EFTR19	Field
	43-3 PTRA19	Field

*Cyanotoxin analysis (HPLC-UV and mcyE gene)*		
Bosque Azul	BAZUL	Strain
San Lorenzo	SNLZ	Strain
Paso del Soldado	PSOLD	Strain

**Table 2:** Abundance of *Planktothrix* populations and physical and chemical characteristics of six natural lakes and one sedimentation pond in a water treatment plant pertaining to the Río Grande de Comitán sub-basin and Montebello Lakes National Park.

Site and mean depth^1^	*Planktothrix agardhii* (cel/ml)	*Planktothrix spiroides* (cel/ml)	*Planktothrix* sp.	Temperature (°C)	pH	Specific conductance (*µ*S cm^−1^)	Dissolved oxygen (mg/L)	Orthophosphate (mg/L)	Ammonium (mg/L)	Nitrate (mg/L)	Fecal Enterococci (UFC)	Soil use
Water treatment plant (PTAR) 3 m	9.01 × 10^8^	1.1 × 10^6^	0	24.8	9.1	684	10.6	38	0.41	5.4	10000	HS
Efluente (EPTR)	0	0	4.1 × 10^4^	24.0	9.1	680	9.0	38.3	0.41	5.45	1000	HS
Balamtetik (BLTK) 1.7 m	4.50 × 10^5^	0	0	23.1	8.5	657	3.4	2.0	0.58	19	317	RA
Chaj Chaj (CHAJ) 5.3 m	9.01 × 10^5^	0	0	24	8.6	620	5.1	1.6	0.22	3.24	126	RA
San Lorenzo (SNLZ)^*∗*^ 11.8 m	1.29 × 10^5^	0	0	25.5	8.7	589	9.7	0.7	0.08	15.5	97	MF
Chilpotrero (CPTO) 2 m	3.38 × 10^4^	0	0	25.2	8.3	530	7.8	0.3	0.07	0.83	25	MF/RA
Paso del Soldado (PSOLD)^*∗*^ 1.5 m	5.0 × 10^3^	0	0	25	7.9	469	4.5	0.1	0.08	0.81	192	MF
Bosque Azul (BAZUL)^*∗*^ 20	2.2 × 10^4^	0	0	23	7.7	387	9.7	0.5	0.76	2.6	ND	MF
La Encantada (LECTD) 27.5	2.5 × 10^4^	0	0	25	8.1	448	7	26	6.71	25	32	MF

^*∗*^Sites collected for cultivation and determination of toxins on July 10, 2023. HS, human settlement; RA, rainfed agriculture; MF, mountain mesophilic forest; ND = not detected.

**Table 3:** Morphological characteristics of the seven populations of *Planktothrix agardhii*, one population of *Planktothrix* sp., and one population of *P. spiroides* in seven natural lakes and two water treatment ponds in the Río Grande de Comitán sub-basin and Montebello Lakes National Park.

Site	Trichomes			Cell length	Apical cell	Cross-wall	Cell color	Aerotopes
	Length	Width	Shape					
Water treatment plant *P. spiroides* *n* = 100	39.2–9.8 64.1 ± 0.02	3.0–6.0 5.1 ± 0.02	Spiral	2.0–3.0 2.1 ± 0.02	Rounded to slightly attenuated	Not constricted	Olive-green to brown	Several small

Efluente *Planktothrix* sp. *n* = 100	36.4–83.15 52.5 ± 26.5	3.3–6.4 4.32 ± 0.15	Straight	1.73–3.8 2.88 ± 0.15	Convex	Slightly constricted	Olive-green	Several small

Balamtetik *P. agardhii* *n* = 117	30.23–211.17 109.13 ± 64.29	3.75–4.96 4.3 ± 0.12	Straight	2.34–3.07 2.69 ± 0.05	Convex with calyptra	Not to slightly constricted	Blue-green to yellow	A few big

Chaj Chaj *P. agardhii n* = 260	27.53–343.33 130.5 ± 85.72	3.7–4.7 4.12 ± 0.09	Straight	2.3–3.16 2.6 ± 0.06	Convex with calyptra	Not to slightly constricted	Blue-green to yellow	A few big

San Lorenzo *P. agardhii* *n* = 150	152.48–420.0 231.77 ± 74.44	3.36–4.34 3.95 ± 0.13	Straight	2.77–3.63 3.07 ± 0.09	Convex with calyptra	Not too slightly constricted	Blue-green to yellow	A few big

Chilpotrero *P. agardhii* *n* = 150	63.39–381.1 245.41 ± 88.39	3.23–4.84 3.73 ± 0.22	Straight	2.79–3.62 3.13 ± 0.05	Convex with calyptra	Not too slightly constricted	Blue-green to yellow	A few big

Paso del Soldado *P. agardhii* *n* = 150	37.74–325.84 134 ± 90.68	2.69–4.22 3.43 ± 0.27	Straight	2.09–3.78 2.69 ± 0.22	Convex with calyptra	Not to slightly constricted	Blue-green to yellow	A few big

La Encantada *P. agardhii* *n* = 135	99.79–250.24 189.39 ± 47.0	2.94–4.62 3.78 ± 0.3	Straight	3.15–4.76 3.66 ± 0.25	Convex with calyptra	Not to slightly constricted	Blue-green to yellow	A few big

Measurements are in µm (mean and standard deviation).

**Table 4:** Mean percent dissimilarities between *Planktothrix* species and the study populations, based on the sequence alignment of the 16S-23S ITS region.

	Species/samples												

1	*P. rubescens*	1	2	3	4	5	6	7	8	9	10	11	12
2	*P. paucivesiculata*	4.7	—	—	—	—	—	—	—	—	—	—	—
3	*P. agardhii*	3.3	4.1	—	—	—	—	—	—	—	—	—	—
4	*P. pseudagardhii*	9.1	10.2	9.4	—	—	—	—	—	—	—	—	—
5	40-1 BLTK19	3.5	3.9	0.2	9.1	—	—	—	—	—	—	—	—
6	41-2 CHAJ19	3.5	3.9	0.2	9.2	—	—	—	—	—	—	—	—
7	B1–22	5.1	5.3	1.6	10.8	1.4	1.4	—	—	—	—	—	—
8	B2–22	5.7	6.1	2.4	11.3	2.2	2.2	3.6	—	—	—	—	—
9	C1–22	4.3	3.9	1.0	9.6	0.8	0.8	2.2	3.1	—	—	—	—
10	C14–22	5.9	5.9	3.0	11.2	3.2	3.2	3.8	5.2	3.2	—	—	—
11	D16–22	4.9	5.3	1.6	10.4	1.4	1.4	2.4	3.5	2.2	3.2	—	—
12	44-2 EFT19	6.1	6.7	5.9	10.4	5.7	5.7	7.1	7.9	5.7	7.8	7.1	—

Values >7.0 are considered strong evidence that the compared groups belong to different species, and values >3.0 are likely different species, while values ∼1.0 likely indicate compared groups belong to the same species.

**Table 5:** Microcystin variants obtained via HPLC-UV from cultures of *Planktothrix agardhii* collected from three lakes in the Río Grande de Comitán sub-basin and Montebello Lakes National Park.

Site	Chla (*μ*g/L)	MC-LR (*μ*g/L)	MC-RR (*μ*g/L)
Bosque Azul	14.020	63.38	26.08
San Lorenzo	21.680	40.08	20.36
Paso del Soldado	25.580	15.86	15.86

**Table 6:** Taxonomic reports and cyanotoxin detection in *Planktothrix* species from Mexico.

Site and water body type	References	Taxa	Taxonomical approach	Field abundance (total phytoplankton)	Toxin genes detected	Total microcystin content (*μ*g/L)
Cuemanco (man-made channels)	Vasconcelos et al. [19]	*Planktothrix agardhii*	Phenotypic and Genotypic	ND	*MycE/ndaf* *MycA* *MycE*	4.9 (ELISA, attributable to *Microcystis aeruginosa*)
	Pineda-Mendoza et al. [18]		Genotypic	ND	ND	ND
	Pineda-Mendoza et al. [63]		Phenotypic and Genotypic	ND	*MycA-Cd*	2.77 (ELISA)

Chapultepec urban lake	Komárek and Komárková-Legnerová [17]	*Planktothrix agardhii*	Phenotypic	ND	ND	ND
	Pineda-Mendoza et al. [64]	*Planktothrix* sp.	Metagenomic	0.037% (OTUs)	ND	1.6–4.5 (ELISA, attributable to *Microcystis* sp)

Xochimilco channel	Komárek and Komárková-Legnerová [17]	*Planktothrix agardhii*	Phenotypic	ND	ND	ND
	Gayosso-Morales et al. [65]	*Planktothrix agardhii*	Phenotypic	Chla 50–350 *μ*g/L		

Ignacio Ramírez dam	Favari et al. [66]	*Oscillatoria agardhii*	Phenotypic	ND	ND	ND

Nabor Carrillo urban Lake	Barrios et al. [67]	*Planktothrix* sp.	Phenotypic	10,678–99,200 Ind/mL	ND	ND

Zumpango Lake	Zamora-Barrios et al. [68]	*Planktothrix agardhii*	Phenotypic	15,236–36,735 Ind/mL	ND	0.1–1.4 (ELISA, with *Microcystis*)

Valle de Bravo dam	Gaytán-Herrera et al. [69]	*Planktothrix agardhii*	Phenotypic	100-10,000 cells/mL	ND	ND
	Alillo-Sánchez et al. [70]		Phenotypic	Until 20,000 cells/mL	ND	0.25–5.5 (ELISA attributable to *Anabaena* spp)

Texcoco Lake	Rea [71]	*Planktothrix agardhii*	Phenotypic	ND	*mcyE-AMT*	0.07 (ELISA, in culture samples)

El Tunal River	Pérez-López [72]	*Planktothrix* sp.	Phenotypic	ND	ND	ND

Montebello lakes	Fernández et al. [26]	*Planktothrix* sp.	Phenotypic	11–33 mm^3^/L	ND	2.0–5.3 (ELISA, attributable to *Raphidiopsis* or *Limnothrix*)

## Data Availability

Data are available from the authors upon reasonable request.
